# Influence of Electron Beam Irradiation on Electrical Insulating Properties of PLA with Soft Resin Added †

**DOI:** 10.3390/polym10080898

**Published:** 2018-08-10

**Authors:** Katsuyoshi Shinyama

**Affiliations:** Department of Electrical and Electronic Engineering, Hachinohe Institute of Technology, 88-1 Ohbiraki, Myo, Hachinohe 031-8501, Japan; shinyama@hi-tech.ac.jp; Tel.: +81-178-25-8295

**Keywords:** polylactic acid, electrical insulating properties, electron beam irradiation

## Abstract

Polylactic acid (PLA), a bioplastic, is a hard resin, and requires softening in order to be used in electric wire sheaths. A soft resin was added to PLA in order to soften it, but this caused the electric breakdown strength (*E*_B_) to decrease. In this study, PLA with soft resin added was irradiated with an electron beam and the influence of the electron beam irradiation on *E*_B_ was examined. The irradiation dose was set at 100 kGy. At 25 °C, the *E*_B_ of PLA with no soft resin added decreased due to the irradiation. On the other hand, the *E*_B_ of PLA with soft resin added showed almost no change due to the irradiation. At 80 °C, the *E*_B_ of PLA with no soft resin added decreased due to the irradiation, as was the case at 25 °C. On the other hand, the *E*_B_ of PLA with soft resin added increased due to the irradiation.

## 1. Introduction

The production of synthetic plastics, including cross-linked polyethylene and epoxy resin, has increased rapidly as a result of developments in the petrochemical industry. These products now dominate all aspects of daily life and industry. Their light weight, ease of processing and viscoelastic features, such as mechanical properties, electric insulation, insulation characteristics, and corrosion resistance, have resulted in synthetic plastics coming into wide use as electric insulation materials. In addition, as consumer electronics and digital devices are quickly becoming more widely available, and with the development of the modern electrical, electronic and networking industries, synthetic plastics will be increasingly used as electric insulation materials. However, most of these devices are disposed of after use and damage the environment, causing a huge societal problem.

Among bioplastics, biodegradable plastics are decomposed in the natural environment by bacterial enzymes to produce water and carbon dioxide. Biodegradable plastic waste can therefore be treated by being buried in the ground. These plastics generate only a small amount of heat during combustion and do not release poisonous materials such as dioxins. This means that using biodegradable plastics instead of synthetic plastics protects the environment.

There are three types of biodegradable plastics: biopolymers, synthetic polymers, and modified natural polymers, each of which is produced by different methods. Synthetic polymers are currently being used.

Synthetic polymers can be further classified into plant-based polymers and mineral-oil-based polymers. The author selected polylactic acid (PLA), which is a plant-based polymer. PLA has greater clarity, strength, rigidity, thermal resistance, and dimensional stability than other biodegradable films, so it can be used in many areas, such as for packaging film or industrial film. In recent years, PLA has been adopted as a FDM 3D printing materials.

Furthermore, as it returns to the earth by decomposing after disposal, products of these new plastics satisfy requirements for both high performance and for resource recycling, meeting the requirements of this ecological era. There is the capacity to produce PLA in large quantities, and it is expected that the materials will enable reductions in costs.

Various studies have been carried out on the use of bioplastics as electric insulation materials, and many of these report on the electrical properties of PLA [1,2,3,4]. However, PLA is not yet actually used as electrical insulation materials. The authors’ own previous studies show that PLA is an excellent electrical insulation material, with high resistivity and dielectric breakdown strength in a temperature range from room temperature to 60 °C, these properties being similar to those of petroleum-derived low-density polyethylene (LDPE) [5,6]. PLA, which is a hard resin, has to be softened in order to be used in electric wire sheaths. The authors have reported on the mechanical and electrical properties of plasticizer-added PLA, which improve its flexibility. However, plasticizers are liquid and tend to bleed out as increasing amounts are added. Therefore, as a result of adding a solid soft resin to PLA, bleed out did not occur but electric breakdown strength (*E*_B_) decreased [7]. In this study, the irradiation of PLA with soft resin added with an electron beam was carried out and the influence of the irradiation on *E*_B_ was examined.

## 2. Experimental Methods

**Figure 1:**
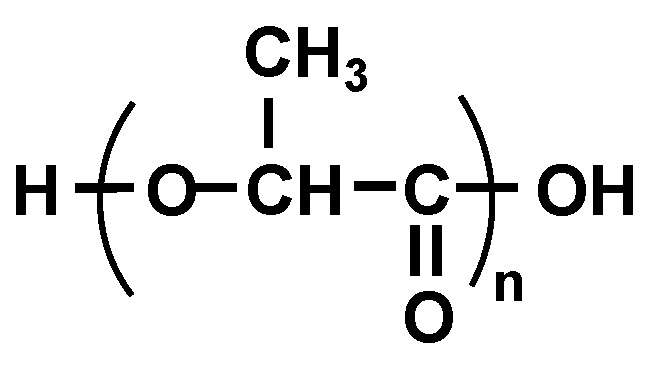
Chemical structural formula of polylactic acid (PLA).

**Figure 2:**
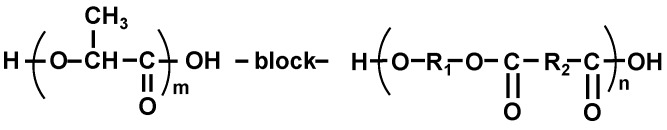
Chemical structural formula of the soft resin.

LACEA, H-100J, produced by Mitsui Chemicals (Tokyo, Japan), Inc. was used as the PLA. Figure 1 shows the chemical structural formula of PLA. PLAMATE PD-350, produced by Dainippon Ink and Chemicals (Tokyo, Japan), Inc., was used as the soft resin. Figure 2 shows the chemical structural formula of the soft resin. It is a copolymer resin of PLA and diol dicarboxylic acid. In Figure 2, R_1_ and R_2_ are alkylene groups. The authors investigated the mechanical properties of PLA with soft resin added. As a result, the break elongations of PLA with 20 wt % of soft resin showed a value comparable to that of LDPE. In addition, the break elongations of PLA with 80 wt % of soft resin added was about three times that of PLA with 20 wt % of soft resin added [7]. Therefore, in this study, the amount of soft resin added to PLA was set to 20%, which indicates break elongations comparable to LDPE, and 80%, which was larger than LDPE in break elongations. In addition, chloroform produced by Kanto Chemical Co., Ltd. (Tokyo, Japan) was used as an organic solvent when soft resin was added to PLA. The sample solution was defoamed for 10 min using a centrifugal defoaming machine and then dried on a hot plate at 60 °C for 30 min by a solution casting method. Following this, the samples were molded in a hot-press at 200 °C and then rapidly cooled. The thickness of the sample was 50 μm for the insulation breakdown test and 100 μm for the tensile test. Table 1 gives the names assigned to the samples of PLA with soft resin added.

Generally, exposing polymer materials to radiation improves their heat resistance, strength, and electrical insulating properties, and this method is currently used to help improve the quality of various materials. In this study, an electron beam that allows a high dose of radiation to be delivered in a short period of time was selected, and using it a dose of radiation was delivered to the PLA with soft resin added. Samples were irradiated with the electron beam in air, using an electron accelerator made available by the Takasaki Advanced Radiation Research Institute (Takasaki, Japan), which belongs to the Japan Atomic Energy Agency. The accelerating voltage and the dose rate were set at 2.0 MV and 80 to 100 Gy/s. When PLA was irradiated with a dose of 100 kGy or more, the collapse reaction was likely to occur, the strength was weakened, and cracks were easily introduced [5]. Therefore, the irradiation dose was set to 100 kGy.

**Figure 3:**
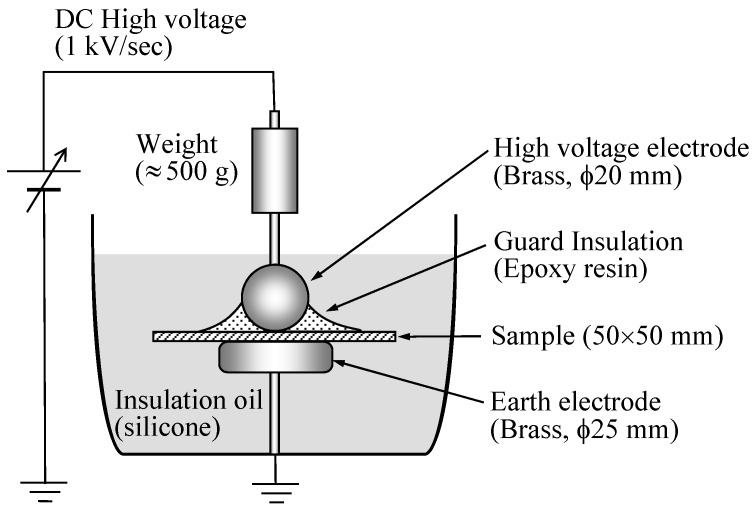
Experimental system for measuring dielectric breakdown strength.

In order to examine electrical properties of irradiated PLA with soft resin added, insulation breakdown tests were carried out. Figure 3 shows the experimental system for measuring dielectric breakdown strength. The breakdown voltages were measured in silicone oil. As will be described later, the glass transition temperature (*T*_g_) for PLA is approximately 50 °C. In addition, the dielectric breakdown strength of PLA decreases at 60 °C or higher [6]. I set the temperature of silicone oil at 25 °C and 80 °C above the *T*_g_ of PLA.

A spherical upper electrode was covered with epoxy resin to prevent creeping discharge, and the lower electrode was in the form of a disk. For the insulation breakdown test, in order to prevent silicone oil from seeping into the gap between the electrode and sample, the epoxy resin surface was smoothened so that the contact between the sample and electrode was as close as possible. A DC high-voltage source was used for the insulation breakdown tests, with DC voltage applied to the samples at a voltage increase rate of 1 kV/s. The breakdown voltage was divided by the thickness of the sample to obtain its electric breakdown strength (*E*_B_). The breakdown voltage values were obtained by performing the corresponding tests at least ten times.

To examine the thermal properties of irradiated PLA with soft resin added, a thermal analysis of the samples was carried out with a heat flux type differential scanning calorimeter (DSC) (Shimadzu Corp., Kyoto, Japan, DSC-60) manufactured. Using α-alumina as a standard specimen, the DSC curves of these samples were observed whilst heating each of them in a nitrogen atmosphere from 20 °C to 200 °C, the temperature being increased at a rate of 10 °C/min.

An X-ray photoelectron spectrometer (XPS) (JEOL Ltd., Tokyo, Japan, JPS-9000) was used for the surface analysis of the irradiated PLA with soft resin added. The X-ray source voltage was set to 10 kV, and the emission current to 5 mA.

**Figure 4:**
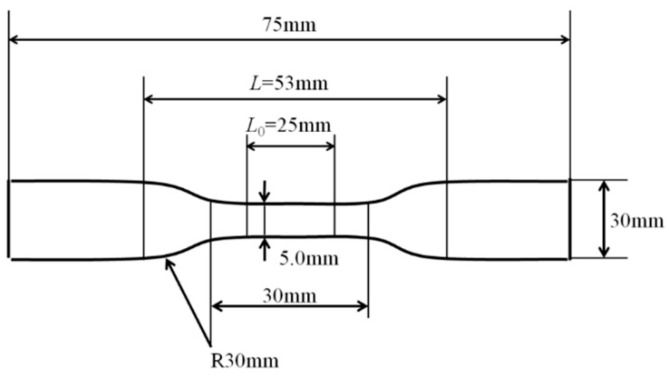
Dimensions of the sample used for tensile test.

In order to investigate the mechanical properties of the irradiated PLA with soft resin added, a tensile test was conducted. Figure 4 shows the dimensions of the sample used for tensile testing. Samples were half of the size specified in the Japanese Industrial Standards (JIS K7113-1995) with a width of 10 mm, an elongated part width of 5 mm, and a length of 75 mm. Two parallel marked lines 25 mm apart were made at the center of the sample. The sample was measured at a speed of 20 mm/min at 25 °C. The tensile test was conducted at least ten times per data item.

## 3. Results and Discussion

**Figure 5:**
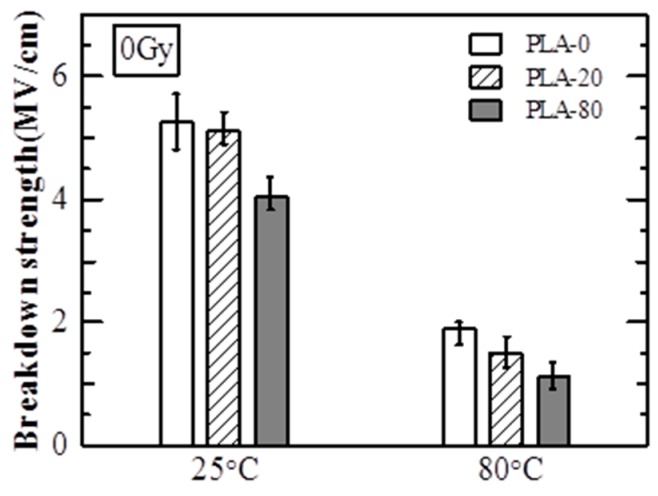
Breakdown strength (*E*_B_) of non-irradiated samples (0 Gy).

Figure 5 shows the *E*_B_ of non-irradiated samples (0 Gy). At 25 °C, the *E*_B_ values of PLA-0, PLA-20, and PLA-80 were approximately 5.3 MV/cm, 5.1 MV/cm, and 4.0 MV/cm, respectively, showing that *E*_B_ decreased as the amount of soft resin added was increased. At 80 °C, the *E*_B_ values of PLA-0, PLA-20, and PLA-80 were approximately 1.9 MV/cm, 1.5 MV/cm, and 1.1 MV/cm, respectively, showing that *E*_B_ decreased as the amount of soft resin added increased, as was the case at 25 °C.

**Figure 6:**
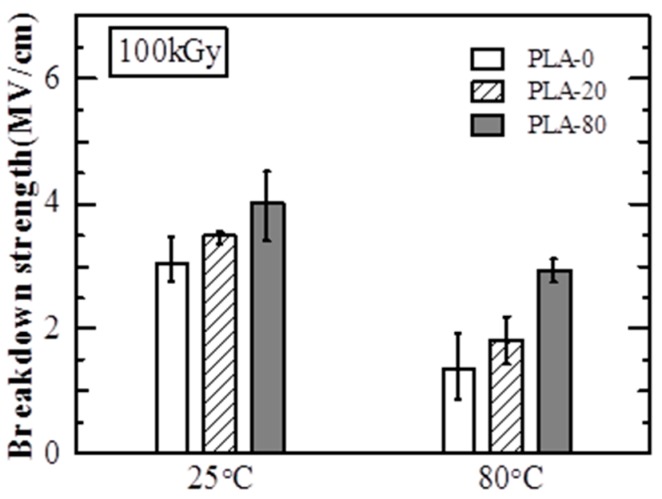
Breakdown strength (*E*_B_) of irradiated samples (100 kGy).

Figure 6 shows the *E*_B_ of irradiated samples (100 kGy). At 25 °C, the *E*_B_ values of PLA-0, PLA-20, and PLA-80 were approximately 3.0 MV/cm, 3.5 MV/cm, and 4.0 MV/cm, respectively. The *E*_B_ values of irradiated PLA-0 and PLA-20 were smaller than those of the non-irradiated samples at 25 °C shown in Figure 5. *E*_B_ increased as the amount of soft resin added increased, and the *E*_B_ value of irradiated PLA-80 was almost the same as that of the non-irradiated sample at 25 °C shown in Figure 5. At 80 °C, the *E*_B_ values of PLA-0, PLA-20, and PLA-80 were approximately 1.4 MV/cm, 1.8 MV/cm, and 2.9 MV/cm, respectively. While the *E*_B_ value of irradiated PLA-0 was smaller than that of the non-irradiated sample at 80 °C shown in Figure 5, the *E*_B_ values of irradiated PLA-20 and PLA-80 were larger than those of the non-irradiated samples at 80 °C shown in Figure 5. At 80 °C, *E*_B_ increased as the amount of soft resin added increased, as was the case at 25 °C.

These results show that while the *E*_B_ of the sample with no soft resin added is decreased due to the electron beam irradiation, that of PLA with soft resin added samples is increased due to the electron beam irradiation.

**Figure 7:**
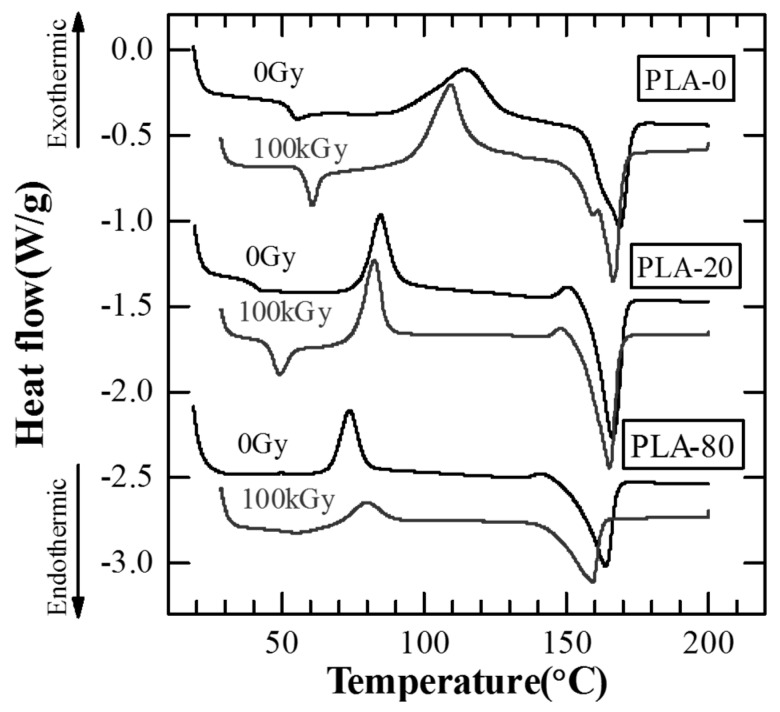
DSC curves for irradiated PLA.

To examine the thermal properties of irradiated PLA with soft resin added, a thermal analysis of the samples was carried out manufactured. Figure 7 shows the DSC curves for irradiated PLA. Table 2 shows the samples’ glass-transition temperatures (*T*_g_), crystallization temperatures (*T*_c_), and melting points (*T*_m_) obtained from the DSC curves.

First, the samples that were not irradiated (0 Gy) by the electron beam are described. *T*_g_ for PLA-0 is approximately 50 °C, and that for PLA-20 is approximately 40 °C, representing a shift to lower temperature compared to PLA-0. *T*_g_ decreased as the amount of soft resin added increased, falling to approximately 20 °C for PLA-80. *T*_c_ for PLA-0 is approximately 115 °C, and that for PLA-20 is approximately 87 °C, representing a downward temperature shift of approximately 30 °C compared to PLA-0. As the amount of soft resin added increased, *T*_c_ for the PLA with soft resin added gradually decreased, becoming approximately 70 °C for PLA-80. *T*_m_ for PLA-0 was approximately 168 °C, *T*_m_ value gradually decreasing as the amount of soft resin added increased, falling to approximately 165 °C for PLA-80. The decrease in *T*_g_, *T*_c_, and *T*_m_ with addition of soft resin is probably due to the soft resin widening the gaps between the molecular chains of the PLA, allowing the molecules to move more freely.

At 25 °C, electron avalanche breakdown was considered to be dominant, as PLA-0 and PLA-20 are at temperatures lower than *T*_g_ and PLA-80 is at around *T*_g_ [6,8,9]. As shown in Table 2, *T*_g_ decreases as the addition amount of soft resin increases, it is considered that *E*_B_ decreases as the addition amount of soft resin increases at 25 °C as shown in the Figure 5.

At 80 °C, since the temperature was higher than *T*_g_ in all samples, it was thought that thermal breakdown and electromechanical breakdown occurred [6,8,9]. It is also known that the Young’s modulus decreases as the added amount of soft resin increases [7], so that thermal breakdown and electromechanical breakdown are likely to occur as the amount of soft resin added increases. Therefore, it is considered that *E*_B_ decreases as the addition amount of soft resin increases at 80 °C as shown in Figure 5.

Next, the irradiation (100 kGy) of the samples by the electron beam is described. *T*_g_ for all samples was raised by the irradiation. Compared to *T*_g_ for non-irradiated samples, that for irradiated PLA-0 and PLA-20 was approximately from 6 °C to 7 °C higher, and that for irradiated PLA-80 was approximately 30 °C higher. *T*_c_ for PLA-0 and PLA-20 was lowered from 5 °C to 6 °C due to the irradiation, while that for PLA-80 was raised approximately 10 °C due to the irradiation. *T*_m_ for PLA-0 and PLA-20 was lowered from 1 °C to 2 °C due to the irradiation, while that for PLA-80 was lowered approximately 7 °C due to the irradiation. These results suggest that electron beam irradiation causes changes in the thermal properties of PLA, and some structural change.

In order to examine the structural change of PLA with soft resin added caused due to the electron beam irradiation, a surface analysis of PLA-0 and PLA-80 was conducted using an XPS, and waveform separation of C1s spectra was performed.

**Figure 8:**
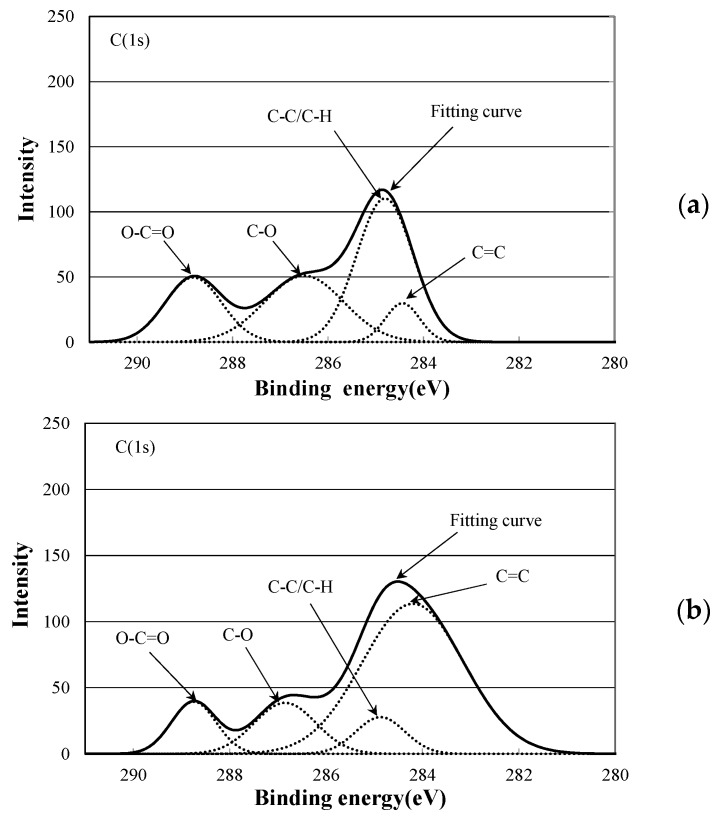
C1s spectrum of PLA-0 at (**a**) 0 Gy; (**b**) 100 kGy.

**Figure 9:**
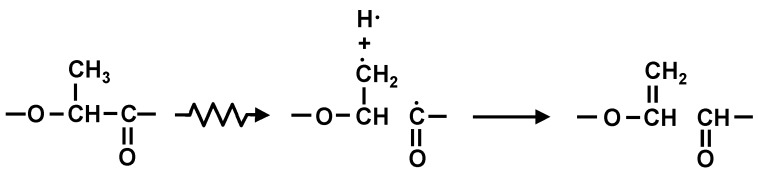
Image of degradation reaction of irradiated PLA-0.

Figure 8 shows C1s spectra of PLA-0. The peak value for C-C or C-H at around 285 eV was approximately 110 before the electron beam irradiation, and decreased by approximately one fourth, to approximately 28, due to the irradiation. This suggests that the irradiation breaks the main and side chains of PLA-0, causing a degradation reaction. On the other hand, the peak value for C=C at around 284 eV was approximately 30 before irradiation, and increased almost fourfold, to approximately 114, due to the irradiation. It is inferred from this that the irradiation breaks the carbon-hydrogen bond present as a side chain, causes hydrogen atom separation, and thus increases C=C as shown in Figure 9. This probably explains the reason for decreases in electric breakdown strength (*E*_B_) of irradiated PLA-0 both at 25 °C and at 80 °C as shown in Figure 5 and Figure 6.

**Figure 10:**
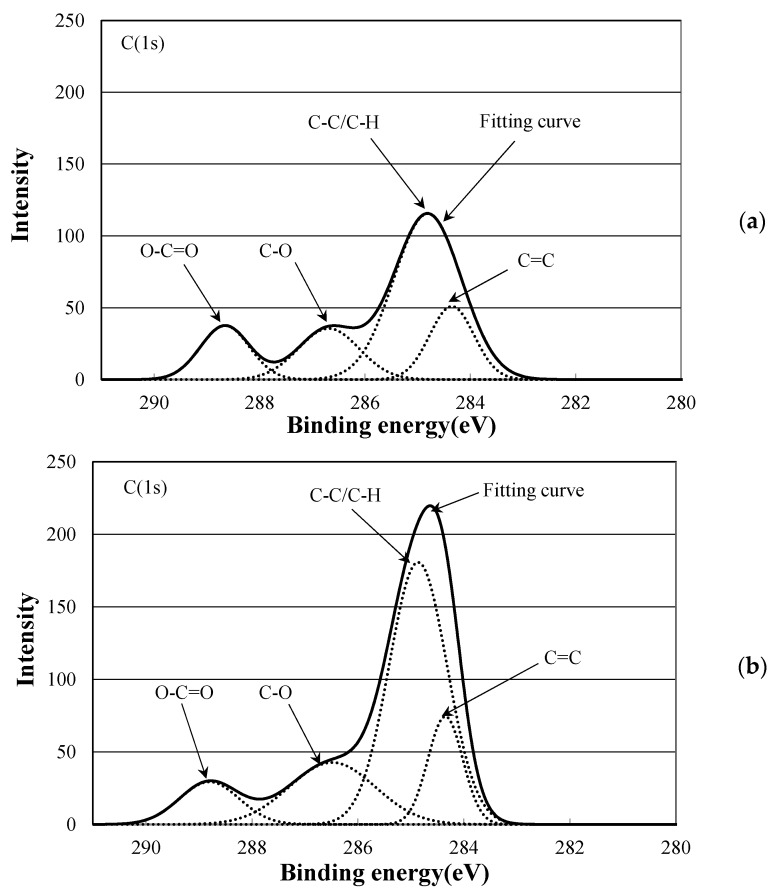
C1s spectrum of PLA-80 at (**a**) 0 Gy; (**b**) 100 kGy.

Figure 10 shows C1s spectra of PLA-80. The peak value for C-C or C-H, at around 285 eV, was approximately 115 before the electron beam irradiation, and was increased approximately 1.6 times, to approximately 181, due to the irradiation. This change is different from that for PLA-0 shown in Figure 8. As Table 2 shows, *T*_g_ for PLA-80 is approximately 20 °C. As PLA-80 was irradiated with an electron beam at room temperature, it is assumed that the sample was rubbery at the time of irradiation.

The effects of electron beam irradiation on diblock and random copolymers of PLA and poly (trimethylene carbonate) (PTMC) have been reported, and as the cleavage of carbon-hydrogen bonds along the backbone of the chains becomes more prevailing as the TMC component increases, it is expected that alkyl radicals are formed in the PTMC segments [10]. The molecular structure of the soft resin used in this study is similar to PTMC. Specifically, in the case of PLA-80 with a large amount of soft resin added, the effect of electron beam irradiation on the chemical structure of the soft resin is presumed to be similar to that of PTMC.

**Figure 11:**
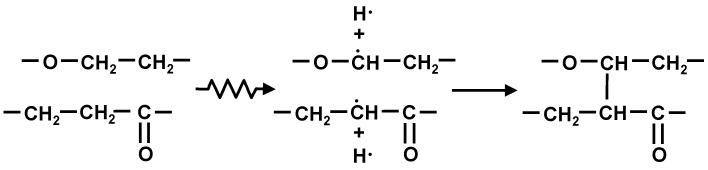
Image of cross-linking reaction of irradiated PLA-80.

It is inferred from this that electron beam irradiation breaks the carbon-hydrogen bonds present as a side change of the soft resin and generates free carbon radicals, and the resultant carbon-atom bonding between molecules promotes a cross-linking reaction as shown in Figure 11.

It has been reported that crosslinking reaction of PLA occurs as a result of adding triallyl isocyanurate (TAIC) and dicumyl peroxide (DCP) to PLA [11,12], and similar reactions may be occurring in this study. On the other hand, the peak value for C=C, at around 284 eV, was approximately 51 before irradiation, and was increased approximately 1.4 times, to approximately 74, due to the electron beam irradiation. This increase was smaller than that for PLA-0 as shown in Figure 8. This probably explains why *E*_B_ was not decreased due to the irradiation for PLA-80 both at 25 °C and at 80 °C as shown in Figure 5 and Figure 6.

**Figure 12:**
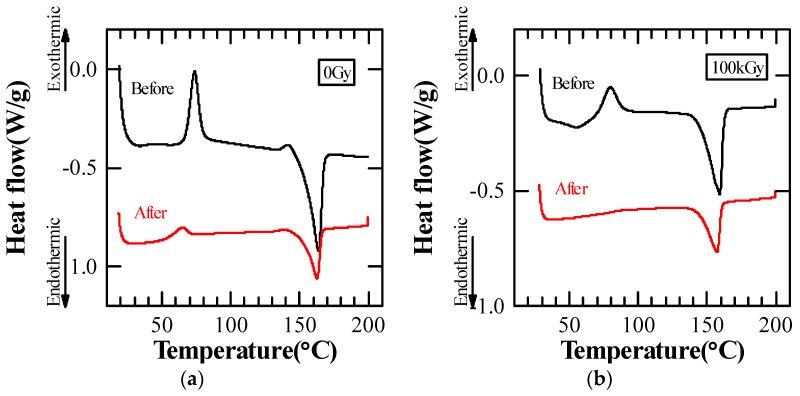
DSC curves of PLA-80 ((**a**) 0 Gy and (**b**) 100 kGy) before and after the dielectric breakdown test at 80 °C.

The thermal properties of PLA-80 before and after the dielectric breakdown test at 80 °C were investigated. Figure 12 shows DSC curves of PLA-80 before and after the dielectric breakdown test at 80 °C. As a result of investigating the DSC curve after conducting the dielectric breakdown test at 80 °C higher than *T*_g_, the peak value for *T*_c_ became smaller in the case of 0 Gy and the value was not seen in the case of 100 kGy. From this result, it is considered that in the case of PLA-80, the crystallization proceeds due to the thermal history during the dielectric breakdown test at 80 °C, and crystallization is more likely to proceed in the irradiated PLA-80. Therefore, as shown in Figure 6, it is considered that *E*_B_ at 80 °C increased as the added amount of soft resin increased.

**Figure 13:**
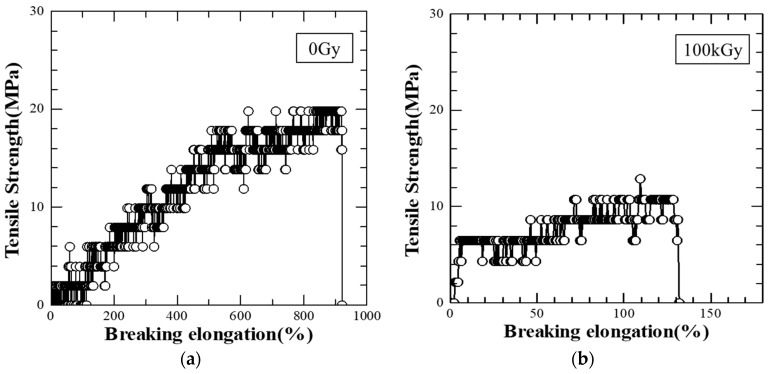
Stress-elongation graph of PLA-80 ((**a**) 0 Gy and (**b**) 100 kGy).

In order to investigate the mechanical properties of the irradiated PLA-80, a tensile test was conducted. Figure 13 shows an example of a stress-strain diagram of the irradiated PLA-80. Table 3 shows average values of tensile strengths at breaking, breaking elongations and Young’s modulus obtained from these figures. The tensile strengths at breaking, breaking elongations and Young’s modulus of PLA-80 were all red due to the electron beam irradiation. It has been reported that as a result of irradiating 33 kGy of electron beam to PLA, stress at break, percentage of elongation, and strain energy decreased [13], and the result is similar to the result of this research.

Therefore, although the crosslinking reaction of PLA with soft resin added does not occur throughout the sample, it is considered that it mainly occurs in the soft resin portion.

## 4. Conclusions

In this study, PLA with soft resin added was irradiated using an electron beam and examined the influence of the electron beam irradiation on the electric breakdown strength (*E*_B_).

For the non-irradiated samples, the electric breakdown strength (*E*_B_) decreased both at 25 °C and at 80 °C as the amount of soft resin added increased. On the other hand, for the irradiated samples, *E*_B_ increased both at 25 °C and at 80 °C as the amount of soft resin added increased.

According to a thermal analysis of PLA, the glass-transition temperature (*T*_g_) was raised due to the electron beam irradiation regardless of whether or not soft resin was added. The crystallization temperature (*T*_c_) was lowered due to the irradiation for the sample with no soft resin added (PLA-0) and for the sample with soft resin added at 20 wt % (PLA-20), but was raised due to the irradiation for the sample with soft resin added at 80 wt % (PLA-80). The melting point (*T*_m_) was lowered due to the irradiation regardless of whether or not soft resin was added. Furthermore, as a result of thermal analysis of PLA-80 before and after the dielectric breakdown test at 80 °C, the peak of *T*_c_ became smaller at 0 Gy and no peak of *T*_c_ was observed at 100 kGy.

A surface analysis of PLA-0 and PLA-80 was conducted using an XPS, and waveform separation of C1s spectra was performed. For PLA-0, the peak value for C-C or C-H was decreased by approximately one fourth due to the electron beam irradiation, and the peak value for C=C was increased almost fourfold by irradiation. For PLA-80, on the other hand, the peak value for C-C or C-H was increased approximately 1.6 times due to the irradiation, and the peak value for C=C was increased approximately 1.4 times due to the irradiation. In the case of the irradiated PLA-80, it is inferred that electron beam irradiation breaks the carbon-hydrogen bonds present as a side change of the soft resin, generating free carbon radicals, and the resultant carbon-atom bonding between molecules promotes a cross-linking reaction.

As a result of examining the mechanical properties of the irradiated PLA-80, the tensile strengths at breaking, breaking elongations and Young’s modulus were all reduced due to the electron beam irradiation.

## Figures and Tables

**Table 1:** Samples of PLA with soft resin added.

Sample	Composition
PLA-0 PLA-20 PLA-80	PLA PLA + 20 wt% soft resin PLA + 80 wt% soft resin

**Table 2:** *T*_g_, *T*_c_ and *T*_m_ obtained from the Figure 7.

Sample	Dose	*T*_g_ (°C)	*T*_c_ (°C)	*T*_m_(°C)
PLA-0	0 Gy	50	115	168
	100 kGy	57	109	166
PLA-20	0 Gy	39	87	166
	100 kGy	45	82	165
PLA-80	0 Gy	20	70	164
	100 kGy	51	80	157

**Table 3:** Average values of tensile strengths at breaking, breaking elongations and Young’s modulus were obtained from the stress-strain diagram of irradiated PLA-80.

Dose	Tensile Strengths at Breaking (MPa)	Breaking Elongations (%)	Young’s Modulus (GPa)
0 Gy	25	971	0.32
100 kGy	12	138	0.05
